# Correlations Between the Microstructural Changes of the Medial Temporal Cortex and Mild Cognitive Impairment in Patients With Cerebral Small Vascular Disease (cSVD): A Diffusion Kurtosis Imaging Study

**DOI:** 10.3389/fneur.2019.01378

**Published:** 2020-01-15

**Authors:** Dongtao Liu, Kun Li, Xiangke Ma, Yue Li, Qiao Bu, Zhenyu Pan, Xiang Feng, Qinglei Shi, Lichun Zhou, Wenli Hu

**Affiliations:** ^1^Department of Neurology, Beijing Chaoyang Hospital, Capital Medical University, Beijing, China; ^2^Department of Radiology, Beijing Chaoyang Hospital, Capital Medical University, Beijing, China; ^3^Department of Neurosurgery, Beijing Chaoyang Hospital, Capital Medical University, Beijing, China; ^4^MR Scientific Marketing, Diagnosis Imaging, Siemens Healthineers China, Beijing, China

**Keywords:** mild cognitive impairment, cerebral small vascular disease, medial temporal cortex, diffusion kurtosis imaging, changes

## Abstract

**Object:** The purpose of our study was to investigate the microstructural changes of the medial temporal cortex in mild cognitive impairment (MCI) patients with cerebral small vascular disease (cSVD) using diffusion kurtosis imaging (DKI) and to examine whether DKI parameters are correlated with MCI.

**Method:** A total of 82 cSVD patients admitted to the Department of Neurology Beijing Chaoyang Hospital, Capital Medical University, were retrospectively enrolled in this study. The Montreal cognitive assessment scale (MoCA) score was used to assess overall cognitive function. According to the presence or absence of MCI, these patients were divided into an MCI group (*n* = 48) and a non-MCI group (*n* = 34). The general clinical data of the two groups were collected and analyzed. The regions of interest (ROIs) in the medial temporal cortex were selected for investigation. The averaged values of DKI parameters were measured in each ROI and compared between the two groups, and the correlations between DKI parameters and MoCA score and between diffusion and kurtosis parameters were examined.

**Results:** Compared to the non-MCI group, MCI patients showed significantly increased mean diffusion (MD) and radial diffusion (RD) and significantly decreased mean kurtosis (MK) in the left hippocampus (*P* = 0.005, 0.006, 0.002, respectively). In the left hippocampus, fractional anisotropy (FA), MK, radial kurtosis (RK), and kurtosis fractional anisotropy (KFA) showed significantly positive correlations with MoCA score (*r* = 0.374, 0.37, 0.392, 0.242, respectively, all *P* < 0.05), while MK and RD were negatively correlated with MoCA score (*r* = −0.227, −0.255, respectively, both *P* < 0.05). In the left parahippocampal region, axial kurtosis (AK) and KFA were positively correlated with MoCA score (*r* = 0.228, 0.282, respectively, both *P* < 0.05), while RK was positively correlated with MoCA score in the right parahippocampal region (*r* = 0.231, *P* < 0.05). Significant correlations of MD with MK, RD with RK, and FA with KFA were observed in the medial temporal cortex (*r* = −0.254, −0.395, 0.807, respectively, all *P* < 0.05) but not of axial diffusion (AD) with AK.

**Conclusion:** The DKI technique can be used to observe microstructural changes of the medial temporal cortex in MCI patients with cSVD. The DKI-derived parameters might be a feasible means of evaluating patients with MCI.

## Introduction

Mild cognitive impairment (MCI) is a condition in which patients demonstrate cognitive impairment with minimal impairment of the instrumental activities of daily living and that does not meet the diagnostic criteria for dementia (1). MCI is common in senior adults, and its prevalence increases with age and lower social/educational status. Patients with MCI are at higher risk of dementia than age-matched controls (2). Studies have shown that the medial temporal cortex is likely to be more vulnerable to MCI and have revealed a 2.2-fold higher volume loss in the hippocampus, 1.8-fold loss in the whole brain, and 1.5-fold loss in the olfactory cortex in MCI patients (3). Takahashi et al. found that a low MoCA score of 22 or less was associated with medial temporal cortex atrophy in patients with amnestic cognitive impairment after stroke (4). The meta-analysis also revealed that the early changes in the olfactory cortex are a good imaging biomarker that can be used to discriminate individuals with MCI from normal control subjects and that a larger degree of atrophy in the olfactory cortex predicts increased disease severity (5). In recent years, many studies have been published on diagnostic applications of diffusion tensor imaging (DTI) (6–10). However, the simplified description of the diffusion process assumed in DTI does not permit complex microstructures to be completely mapped because the cellular components and structures hinder and restrict the diffusion properties of water molecules. These limitations can be partially overcome by DKI, and DKI parameters have been found to be very sensitive in identifying some alterations that characterize many neurological diseases (11, 12). These changes are appreciable with DKI even before any imaging findings through conventional imaging and in a better way than with conventional DTI (13). However, few DKI study results are available with which to comprehensively investigate the changes in the medial temporal cortex in patients with MCI. This study aimed to identify the early microstructural alterations in the medial temporal cortex in MCI patients with cSVD by DKI and further examine the relationship between these parameters and MoCA score, which may provide neuroimaging evidence for the evaluation of MCI patients.

## Methods

### Subjects

We retrospectively collected 82 patients with cSVD who were admitted to the Department of Neurology, Beijing Chaoyang Hospital, Capital Medical University from January to December 2018, and the diagnosis was confirmed by conventional MRI scan (including MRA) of the head (14). Inclusion criteria were: age of ≥50 years, cranial MRI confirmed the presence of cSVD (15), evaluation of daily life showed no functional disability, evaluation of overall cognitive function performed using the MoCA score. Exclusion criteria were: patients with severe medical diseases, such as heart diseases, liver diseases, renal failure, tumors, or other systemic diseases; patients with severe neurological diseases, such as white matter lesions unrelated to vascular diseases, tumor, Parkinson's disease, and brain trauma; patients with severe neuropsychological disorders, mental disease, or medicated with drugs affecting cognition within the prior 24 h; patients with contradictions to MRI or who were unable to receive cranial MRI.

### Cognitive Function and Neuropsychological Assessment

All patients were assessed on the neuropsychological scale at admission. We use the MoCA score to assess overall cognitive function, which included attention and concentration, executive function, memory, language, visual-spatial structure skills, abstract thinking, calculation, and orientation. According to their social/educational status, those in the illiterate group with ≤13 points, in the primary school group with ≤19 points, and in the junior high school and above group with ≤24 points were considered to have objective cognitive impairment (16). The 24 stems of the Hamilton Depression Scale (HAMD) and the Hamilton Anxiety Scale (HAMA) were also performed to assess the severity of depressive or anxiety disorders. All subjects underwent routine blood biochemical tests and glycosylated hemoglobin and serum homocysteine tests. The baseline data collected for all subjects were age, gender, education, hypertension, diabetes mellitus, hyperlipidemia, smoking, and drinking history.

### General Criteria for MCI

First, the patient is neither normal nor demented; second, there is evidence of cognitive deterioration, shown by either an objectively measured decline over time and/or subjective report of decline by self and/or informant in conjunction with objective cognitive deficits; third, activities of daily living are preserved, and complex instrumental functions are either intact or minimally impaired (17).

## MRI Data Collection

### MRI Scanning

All patients were scanned on a 3 Tesla whole-body MR system (MAGNETOM Skyra, Siemens Healthcare, Erlangen, Germany) with a 20-channel phased-array head coil. The head was fixed with a sponge mat. T1-weighted images (T1WI) were scanned using a 3D magnetization-prepared rapid acquisition gradient echo (MPRAGE). The sequence parameters were set as follows: repetition time (TR) = 2,300 ms, inversion time (TI) = 900 ms, echo time (TE) = 89 ms, flip angle (FA) = 8°, field-of-view (FOV) = 240 × 240 mm, voxel size = 0.9 mm isotropic, parallel acceleration factor (PAT) = 2, and acquisition time = 5 min 21 s. The diffusion imaging was performed using spin-echo echo-planar imaging (SE-EPI) and was scanned in two blocks. The parameters of the first block were TR = 7,700 ms, TE = 89 ms, imaging matrix = 74 × 74, FOV = 222 × 222 mm, number of slices = 50, slice thickness = 3 mm, b = 0, 1,000, 2,000 s/mm^2^, 1 average, 30 gradient direction, PAT = 2, and the acquisition time was 8 min 14 s. The parameters of the second block were the same as for the first except only b = 0 s/mm^2^ was used, 9 average, and acquisition time was 1 min 34 s. The total scan time of the diffusion scan was 9 min 48 s.

### Processing of DKI Data

Two radiologists (KL with 10 years' experience in neuroimaging, ZP with 20 years' experience in neuroimaging) viewed the images. The medial temporal cortex mainly includes the hippocampus, and olfactory and parahippocampal regions, and the label indices of 21, 22, 37, 38, 39, 40 were selected as ROIs according to the Anatomical Automatic Labeling (AAL) template (18). The scanned diffusion-weighted images were first transformed to NII file format using the *dcm2nii* tool and then supplied to the diffusional kurtosis estimator (DKE) to generate DKI parameter maps. However, during the acquisition, to reduce the eddy current (EC) effect, we applied the vendor-provided EC sensitivity reduction and dynamic field correction option in the protocol. The T1W images acquired by MP-RAGE were supplied to the SPM12 toolbox (19). The AAL template was non-linearly registered to T1W images, and the AAL labels were aligned to the T1W image space using the generated wrapping field and transformation matrix. The DWI images (*b* = 0 s/mm^2^) were rigidly aligned to the T1WI space, and the transformed matrix was applied onto the DKI parameter maps. The average values of MD, AD, RD, FA, MK, AK, RK, and KFA in these segmented ROIs were then automatically calculated using MATLAB (2017a, The MathWorks, Inc., Natick, MA). Although, in gray matter, due to its near isotropic diffusion, the independent parameters were considered to contain only MD and MK (12), we presented the preliminary results in this study for all parameters including the directional ones to provide a comprehensive perspective with potential findings. MK is calculated as the average of the kurtosis along all directions of diffusion gradients (20), and AK, RK, and KFA are calculated similarly to AD, RD, and FA, which are of interest for white matter bundles since they give additional information on the axonal and myelin integrity (21).

### Statistical Analyses

Statistical analyses were performed using SPSS (version 22.0, IBM Corp., Armonk, NY). The one-sample Kolmogorov–Smirnov test was applied to test the normality of the data distribution. Data were expressed as mean ± standard deviation ($\bar{X}$ ± SD) when normality assumptions were satisfied. Otherwise, data were expressed in terms of quartile. The independent sample *t*-test, Mann–Whitney *U*-test, or the χ^2^-tests were used appropriately for comparison between the two groups. Multivariate logistic regression was applied to determine the risk factors for patients with MCI. We corrected multiple comparisons using the Šídák-Bonferroni method, and the corrected *P*-value was statistically significant when *P* < 0.0083 (0.05/6 = 0.0083). Correlation between the MoCA score and the DKI diffusivity and kurtosis parameters were analyzed. Pearson correlation analysis was applied when normality assumptions were satisfied; otherwise, Spearman correlation analysis was used. A value of *P* < 0.05 was considered statistically significant.

## Results

### General Characteristics and Cognitive Functions

Of the 82 patients, 47 were male and 35 were female. The age ranged from 50 to 88 years, with a median age of 64 (59, 69) years old. The length of education was from 0 to 18 years, and the median length was 8 (8, 11) years. According to the presence or absence of MCI, the 82 patients were divided into an MCI group (48 cases) and a non-MCI group (34 cases). There were 28 males and 20 females in the MCI group, their ages ranged from 50 to 88 years old, with a median age of 64 (60, 71) years old, their education period was from 0 to 18 years, and the median duration of education was 8 (7, 10) years. There were 19 males and 15 females in the non-MCI group, their ages ranged from 50 to 78 years old, with a median age of 63 (57, 67) years old, their duration of education was from 8 to 11 years, and the median duration of education was 9.5 (8, 11) years. There were no significant differences in gender, age, and years of education between the two groups (*P* > 0.05).

The risk factors of cerebrovascular disease (such as hypertension, diabetes mellitus, hyperlipidemia, and history of smoking and drinking), the blood test results (such as total cholesterol, serum glucose, serum homocysteine, etc.), the MoCA score, and the HAMA and HAMD score were also comparable between the groups. MCI patients had evident cognitive impairment and shared significant reductions in MoCA score (*P* < 0.01). Patients with non-MCI had more type 2 diabetes mellitus. However, there was no significant difference between the two groups after multivariate logistic regression was applied (see Table 1). We have also evaluated the enlarged perivascular space (EPVS) and white matter hyperintensities (WMHs) according to cranial MRI (see Supplementary Figures 1, 2). We found patients with MCI had more severe total WMHs, however, there were no significant differences between the two groups in the severity of EPVS. Logistic regression was performed to determine risk factors for patients with MCI, and found that the severity of WMHs was an independent risk factor for MCI patients (see Supplementary Tables 1, 2).

**Table 1:** General characteristics and cognitive function of MCI and non-MCI patients.

	**MCI group *N* = 48**	**Non-MCI group *N* = 34**	**U/x^**2**^**	***P***
Age (year)	64 (60, 71)	63 (57, 67)	−0.862	0.389
Sex (male)	28 (58.3%)	19 (55.9%)	0.049	0.825
Duration of education (year)	8 (7, 10)	9.5 (8, 11)	−1.708	0.088
HAMD	23 (47.9%)	14 (41.2%)	0.365	0.546
HAMA	19 (39.6%)	13 (38.2%)	0.015	0.902
Hyperlipidemia	27 (56.3%)	15 (44.1%)	1.173	0.279
DM	10 (20.8%)	14 (41.2%)	3.979	0.046^*^
Hypertension	33 (68.8%)	21 (61.8%)	0.432	0.511
History of drinking	13 (27.1%)	11 (32.4%)	1.794	0.498
History of smoking	18 (37.5%)	14 (41.2%)	0.138	0.934
Glucose (mmol/L)	5.3 (4.9,6.5)	5.3 (4.7, 7.2)	−0.108	0.914
UA (umol/L)	294 (236, 381)	283 (253, 346)	−0.376	0.707
CR (umol/L)	69.2 (63.4, 84.9)	67.5 (56.7, 75.7)	−1.633	0.102
TC	4.4 (3.6, 4.9)	4.5 (3.9, 5.0)	−0.734	0.463
TG	1.5 (1.1, 1.9)	1.3 (0.9, 1.9)	−0.932	0.351
LDL-C	2.3 (1.9, 2.8)	2.4 (2.−0, 2.8)	−0.438	0.662
LPa	174 (71, 385)	135 (54, 182)	−1.412	0.158
HCY	14.3 (12, 16.5)	12.3 (10.8, 15.3)	−1.823	0.068
Glycated hemoglobin	6 (5.8, 6.8)	6.4 (5.8, 6.8)	−0.972	0.331
MoCA	21 (19, 22)	27 (5, 28)	−6.983	0.000^**^

*DM, Diabetes mellitus; UA, Uric acid; Cr, Creatinine; TC, Total cholesterol; TG, Triglyceride; LDL-C, Low-density lipoprotein cholesterol; Lpa, Lipoprotein a; HCY, Homocysteine; MoCA, Montreal Cognitive Assessment; MCI, mild cognitive impairment*.

* P < 0.05,

** *P < 0.01*.

### Comparison of DKI Parameters in the Medial Temporal Cortex Between the MCI and Non-MCI Groups

Compared to the non-MCI group, the MCI group showed significantly increased MD and RD (*P* = 0.005, 0.006, respectively) and significantly decreased FA, AK, MK, RK, and KFA in the left hippocampal region (*P* = 0.017, 0.01, 0.002, 0.016, and 0.023, respectively). In the right olfactory region, AK and MK were significantly lower in patients with MCI (*P* = 0.027 and 0.037, respectively), while in the left parahippocampal region, AK was significantly lower in patients with MCI (*P* = 0.044). No parameters were found to be significantly different between the two groups in the left olfactory, right hippocampus, and right parahippocampal regions. The Šídák-Bonferroni method was applied for multiple comparisons, and MD, RD, and MK still remained statistically significantly different in the left hippocampal region (*P* = 0.005, 0.006, and 0.002, respectively; see Tables 2–4).

**Table 2:** Comparison of DKI parameters in the hippocampus between patients in the MCI and non-MCI groups.

**Group**	**MCI group** ***N*** **= 48**		**Non-MCI group** ***N*** **= 34**		***t/U*-Value^a^**	***P*-Value^a^**	***t/U*-Value^b^**	***P*-Value^b^**
	**Left**	**Right**	**Left**	**Right**				
AD	1.99 (1.82, 2.29)	1.80 (1.63, 2.07)	1.96 (1.77, 2.06)	1.67 (1.58, 1.90)	644.0	0.105	628.0	0.077
MD	1.79 ± 0.30	1.51 (1.30, 1.80)	1.63 ± 0.22	1.37 (1.25, 1.53)	620.0	0.065	5.51	0.005
RD	1.65 ± 0.29	1.36 (1.17, 1.63)	1.48 ± 0.23	1.24 (1.11, 1.40)	645.0	0.108	3.94	0.006
FA	0.16 (0.14, 0.19)	0.21 ± 0.06	0.18 (0.16, 0.24)	0.22 ± 0.07	0.44	0.72	563.5	0.017
AK	0.63 ± 0.05	0.65 ± 0.06	0.66 ± 0.04	0.68 ± 0.06	0.00	0.08	3.28	0.01
MK	0.70 ± 0.07	0.74 ± 0.08	0.74 ± 0.07	0.75 ± 0.09	0.15	0.37	0.00	0.002
RK	0.74(0.70, 0.80)	0.83 ± 0.13	0.79(0.74, 0.92)	0.84 ± 0.15	0.37	0.67	559.0	0.016
KFA	0.26 ± 0.05	0.31 ± 0.05	0.29 ± 0.05	0.32 ± 0.07	0.53	0.30	0.16	0.023

*DKI, Diffusion kurtosis imaging; MCI, Mild cognitive impairment; AD, Axial diffusion; MD, Mean diffusion; RD, Radial diffusion; FA, Fractional anisotropy; AK, Axial kurtosis; MK, Mean kurtosis; RK, Radial kurtosis; KFA, Kurtosis fractional anisotropy*.

a *The right-side group test value and P-value*.

b *The left-side group test value and P-value*.

**Table 3:** Comparison of DKI parameters in the olfactory region between patients in the MCI and non-MCI groups.

**Group**	**MCI group** ***N*** **= 48**		**Non-MCI group** ***N*** **= 34**		***t/U*-Value^a^**	***P*-Value^a^**	***t/U*-Value^b^**	***P*-Value^b^**
	**Left**	**Right**	**Left**	**Right**				
AD	1.70 ± 0.43	1.69 ± 0.39	1.66 ± 0.36	1.66 ± 0.37	0.21	0.77	0.32	0.65
MD	1.47 ± 0.41	1.45 ± 0.35	1.46 ± 0.35	1.43 ± 0.37	0.19	0.77	0.14	0.85
RD	1.38 ± 0.37	1.33 ± 0.34	1.33 ± 0.31	1.30 ± 0.36	0.44	0.67	0.22	0.53
FA	0.15 ± 0.04	0.16 ± 0.04	0.15 ± 0.03	0.17 ± 0.05	0.82	0.24	0.41	0.50
AK	0.70 (0.65, 0.76)	0.68 (0.64, 0.73)	0.71 (0.67, 0.79)	0.72 (0.67, 0.78)	581.0	0.027	726.5	0.40
MK	0.72 (0.66, 0.77)	0.71 (0.66, 0.76)	0.73 (0.69, 0.80)	0.78 (0.69, 0.83)	594.0	0.037	712.0	0.328
RK	0.72 (0.65, 0.82)	0.73 (0.66, 0.82)	0.74 (0.70, 0.80)	0.80 (0.68, 0.87)	637.0	0.092	706.0	0.301
KFA	0.28 (0.24, 0.32)	0.29 (0.25, 0.34)	0.29 (0.26, 0.32)	0.31 (0.26, 0.40)	734.0	0.44	728.0	0.408

*DKI, Diffusion kurtosis imaging; MCI, Mild cognitive impairment; AD, Axial diffusion; MD, Mean diffusion; RD, Radial diffusion; FA, Fractional anisotropy; AK, Axial kurtosis; MK, Mean kurtosis; RK, Radial kurtosis; KFA, Kurtosis fractional anisotropy*.

a *The right-side group test value and P-value*.

b *The left-side group test value and P-value*.

**Table 4:** Comparison of DKI parameters in the parahippocampus between patients in the MCI and non-MCI groups.

**Group**	**MCI group** ***N*** **= 48**		**Non-MCI group** ***N*** **= 34**		***t/U*-Value^a^**	***P*-Value^a^**	***t/U*-Value^b^**	***P*-Value^b^**
	**Left**	**Right**	**Left**	**Right**				
AD	2.20 ± 0.37	1.90 ± 0.25	1.14 ± 0.27	1.93 ± 0.34	3.72	0.75	1.52	0.44
MD	1.92 ± 0.35	1.65 ± 0.23	1.88 ± 0.25	1.68 ± 0.33	4.22	0.73	1.58	0.57
RD	1.78 ± 0.34	1.53 ± 0.23	1.73 ± 0.25	1.54 ± 0.32	4.66	0.79	1.16	0.48
FA	0.15 (0.13, 0.17)	0.15 (0.14, 0.18)	0.16 (0.14, 0.17)	0.16 (0.14, 0.18)	771.0	0.762	688.0	0.228
AK	0.63 ± 0.08	0.66 (0.62, 0.68)	0.65 ± 0.04	0.67 (0.64, 0.71)	663.0	0.15	9.15	0.044
MK	0.69 ± 0.10	0.69 ± 0.07	0.71 ± 0.05	0.71 ± 0.06	0.14	0.19	6.32	0.175
RK	0.72 (0.66, 0.78)	0.73 ± 0.10	0.74 (0.71, 0.82)	0.76 ± 0.08	0.32	0.27	630.0	0.08
KFA	0.23 ± 0.04	0.27 ± 0.06	0.25 ± 0.03	0.27 ± 0.06	0.22	0.94	0.68	0.087

*DKI, Diffusion kurtosis imaging; MCI, Mild cognitive impairment; AD, Axial diffusion; MD, Mean diffusion; RD, Radial diffusion; FA, Fractional anisotropy; AK, Axial kurtosis; MK, Mean kurtosis; RK, Radial kurtosis; KFA, Kurtosis fractional anisotropy*.

a *The right-side group test value and P-value*.

b *The left-side group test value and P-value*.

### Spearman Correlations With DKI Parameters and MoCA Score

**Figure 1:**
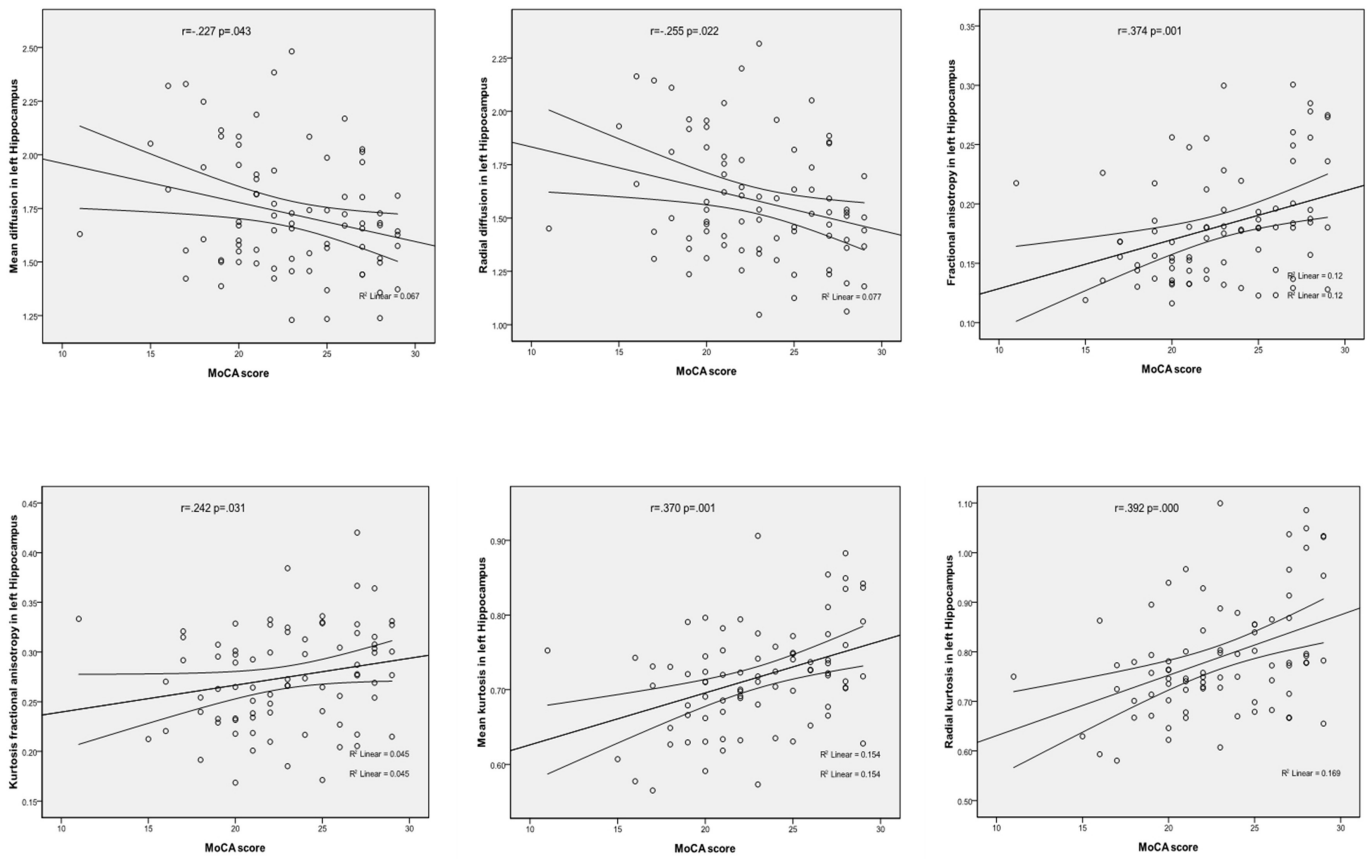
Correlations between DKI parameters and MoCA score in left Hippocampus.

In the left hippocampal region, FA, MK, RK, and KFA were positively correlated with MoCA score (*r* = 0.374, 0.370, 0.392, and 0.242, respectively, all *P* < 0.05), while MD and RD were negatively correlated with MoCA score (*r* = −0.227 and −0.255, respectively, both *P* < 0.05). In the left parahippocampal region, AK and KFA were positively correlated with MoCA score (*r* = 0.228 and 0.282, respectively, both *P* < 0.05), and RK was positively correlated with MoCA score in the right parahippocampal region (*r* = 0.231, *P* < 0.05), while the other parameters had no correlation with MoCA score (detailed Spearman coefficients are summarized in Table 5 and Figure 1).

**Table 5:** Spearson's correlations of DKI parameters with MoCA score.

**MoCA**	**Brain region**	**AD**	**MD**	**RD**	**FA**	**AK**	**KFA**	**MK**	**RK**
	Olfactory_R	−0.035	−0.029	−0.036	0.134	0.157	0.161	0.137	0.161
	Olfactory_L	−0.033	0.000	−0.034	−0.009	−0.010	0.097	−0.078	−0.034
	Hippocampus_R	−0.079	−0.138	−0.145	0.109	0.106	0.095	0.173	0.154
	Hippocampus_L	−0.147	−0.227^*^	−0.255^*^	0.374^**^	0.220	0.242^*^	0.370^**^	0.392^**^
	Parahippocampal_R	−0.074	−0.077	−0.086	0.143	0.212	0.097	0.218	0.231^*^
	Parahippocampal_L	−0.131	−0.122	−0.134	0.152	0.228^*^	0.282^*^	0.180	0.161

*R, Right; L, Left; AD, Axial diffusion; MD, Mean diffusion; RD, Radial diffusion; FA, Fractional anisotropy; AK, Axial kurtosis; MK, Mean kurtosis; RK, Radial kurtosis; KFA, Kurtosis fractional anisotropy; MoCA, Montreal cognitive assessment scale*.

* P < 0.05,

** *P < 0.01*.

### Pearson Correlations Between Diffusivity and Kurtosis Parameters

**Figure 2:**
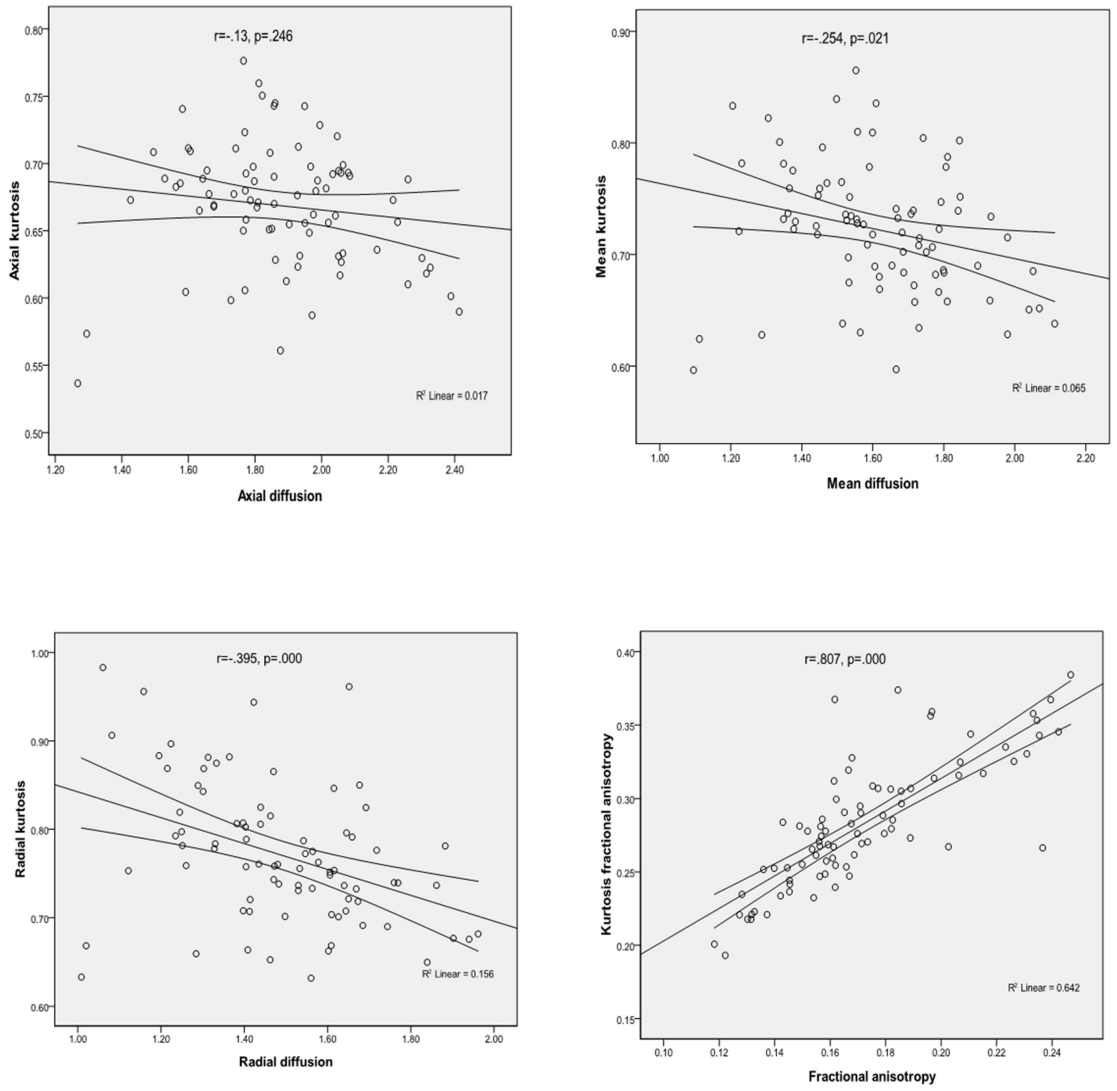
Correlations between diffusivity and kurtosis parameters from all ROIs.

Considering the values of parameters obtained from all patients for the medial temporal regions, there were significant positive correlations between FA and KFA (*r* = 0.807, *P* < 0.001), and RD was found to negatively correlate with RK (*r* = −0.395, *P* < 0.001). Similar inverse correlation was observed between MD and MK (*r* = −0.254, *P* = 0.021), but there were no significant correlations between the AD and AK parameters (Figure 2 presents the correlations mentioned above).

## Discussion

Our study mainly found that compared to the non-MCI group, the MCI group showed significantly increased MD and RD and significantly decreased MK in the left hippocampal region. In the left hippocampal region, FA, MK, RK, and KFA were significantly positively correlated with MoCA score, while MD and RD were significantly negatively correlated with MoCA score. In the left parahippocampal region, AK and KFA were significantly positively correlated with MoCA score, while RK was significantly positively correlated with MoCA score in the right parahippocampal region. With the exception of AD and AK, significant correlations were observed between the other three diffusion and kurtosis parameters in the medial temporal cortex.

In our study, the results showed that compared to non-MCI patients, the values of MK in the left hippocampal region was significantly lower in MCI patients, while the values of MD and RD were significantly increased. Consistent with our results, earlier studies comparing MCI, Alzheimer's disease, and controls also found decreased values of MK in MCI and Alzheimer's disease (12, 22). Falangola et al. demonstrated that MCI and Alzheimer's disease patients showed statistically significant differences in kurtosis parameters in selected brain regions (segmented prefrontal white matter, prefrontal oval, genu of the corpus callosum, anterior corona radiate, segmented temporal white matter, temporal oval, and hippocampus) compared to controls (22). Ryu et al. also found that patients with subjective memory impairment (SMI) exhibited DTI changes (lower FA and higher MD in SMI) in the hippocampal body and olfactory white matter compared to controls (23). The decrease of MK and elevated MD and RD suggests a change in the gray matter microstructure in the medial temporal cortex. This may be due to the loss of neuron cell bodies, synapses, and dendrites, which would increase the extracellular space and result in elevated mean diffusivity and radial diffusivity.

Our study also found that in the left hippocampal region, FA, MK, RK, and KFA were positively correlated with MoCA score, while MD and RD were negatively correlated with MoCA score. In the left parahippocampal region, AK and KFA were positively correlated with MoCA score, and in the right parahippocampal, RK was positively correlated with MoCA score, while the other parameters were observed have no correlation with MoCA score. NJ G etc. found no significant correlations between MMSE score and any of the kurtosis parameters in the gray matter of the temporal cortex (12); this may have been due to the small number of cases, as their study had only 18 Alzheimer's disease patients and 12 MCI patients. We also found that, with the exception of AD and AK, significant correlations were observed between kurtosis and diffusivity parameters (between MK and MD, between FA and KFA, and between RK and RD), which is consistent with the study of NJ G. The results suggested that the changes in diffusivity were accompanied by a change in diffusional non-gaussianity, and kurtosis parameters were suggested to be at least complementary to, if not more sensitive than diffusivity parameters for detecting microstructural changes in the medial temporal cortex.

In addition, our study revealed bilateral asymmetry in the microstructural changes of the medial temporal cortex in patients with MCI. Compared to non-MCI patients, the microstructural changes in the left hippocampus were more obvious than in the right in MCI patients. The possible reason for this is that, in normal people, the hippocampal cortex shows asymmetry. A meta-analysis of asymmetry of the hippocampus and amygdala revealed that both the hippocampus and the amygdala are reliably asymmetrical structures in normal adults, with larger right hippocampal and right amygdala volumes (24). This finding was also supported by Yue et al. in a study of subjective cognitive decline among community-dwelling Chinese (25). De Toledo-Morrell et al. found that the right olfactory cortex may be more vulnerable to the aging process than the left because it was smaller in elderly subjects (26). A recent longitudinal study showed that a smaller thickness in the right olfactory region predicted the onset of symptoms (27). Therefore, we speculated that the left hippocampus is smaller than the right, and the left hippocampal microstructural changes are more vulnerable in MCI patients.

Our study also has several limitations. First, the relatively small sample size may have contributed to the significant group differences. Second, our study lacks a normal healthy control group and long-term follow-up, which has a certain impact on the results of the study. Third, due to the small number of MCI patients in this study, we did not further analyze the subtypes of MCI. Fourth, although interesting findings regarding kurtosis parameters were observed in the medial temporal cortex, their underlying pathophysiological significance must be examined in further studies.

## Conclusion

It is feasible to use DKI to observe the microstructural changes of the medial temporal cortex in MCI patients with cSVD. Compared to the non-MCI group, DKI-derived parameters of the medial temporal cortex were significantly different in the MCI group. Furthermore, some of the DKI parameters showed heterogeneous patterns of correlations with the clinical evaluation score of MCI patients, which might provide insights into the imaging evaluation of MCI patients with cSVD.

## Data Availability Statement

The datasets generated for this study are available on request to the corresponding author.

## Ethics Statement

This study was approved by the Ethics Committee of Affiliated Beijing Chaoyang Hospital of Capital Medical University, and informed consent was obtained from each patient before study. The patients/participants provided their written informed consent to participate in this study.

## Author Contributions

DL, KL, YL, QB, ZP, and XM contributed to the preparation of the manuscript and data collection. XF and QS contributed to data analysis and interpretation. LZ and WH contributed to the experimental design and manuscript revision.

### Conflict of Interest

The authors declare that the research was conducted in the absence of any commercial or financial relationships that could be construed as a potential conflict of interest.
