# Supplementary material for: Correlations Between the Microstructural Changes of the Medial Temporal Cortex and Mild Cognitive Impairment in Patients With Cerebral Small Vascular Disease (cSVD): A Diffusion Kurtosis Imaging Study
Source: Front Neurol. 2020 Jan 15;10:1378. doi: 10.3389/fneur.2019.01378 (PMC6974677; doi:10.3389/fneur.2019.01378)
Supplement: Supplementary file 1 [file Data_Sheet_1.docx]

**Supplementary**

**WMHs and EPVS**

White matter hyperintensities (WMHs) and Enlarged perivascular space (EPVS) were typical imaging manifestations of cSVD ([1](#_ENREF_1)). Assessments of EPVS and WMHs were performed by two experienced neurologists (K. L and Z. P). Discrepancies were resolved by consensus. The k statistic of the intra-rater and inter-rater agreement was 0.85 or above, indicating good reliability.

EPVS was defined as CSF-like signal intensity lesions of round, ovoid, or linear shape of <3 mm and located in areas supplied by perforating arteries. We distinguished lacune from EPVS by their larger size (>3 mm), spheroid shape and surrounding hyperintensities on fluid attenuated inversion recovery (FLAIR). Basal ganglia enlarged perivascular space (BG-EPVS) and white matter enlarged perivascular space (WM-EPVS) were separately assessed. The degree of EPVS severity was scored using a scale that was derived from previous studies: 0=no EPVS, 1=≤ 10 EPVS, 2=11 to 20 EPVS, 3=21 to 40 EPVS, and 4=>40 EPVS. The number referred to the highest number of EPVS on one side of the brain ([2](#_ENREF_2)). We classified EPVS into three categories: mild=score 0 or 1; moderate=score 2; and severe=score 3 or 4 (Fig. 1) ([3](#_ENREF_3)). WMHs were scored using the Fazekas scale. A detailed description of these assessments has been previously described. Periventricular white matter hyperintensities (P-WMHs) and deep white matter hyperintensities (D-WMHs) were evaluated separately and summed as Fazekas scores. The degree of WMHs were rated by Fazekas scores (mild: 0 to 2; moderate: 3 to 4; severe: 5 to 6) (Fig. 2) ([4](#_ENREF_4)).

Our study found that MCI patients had more severe total WMHs. However, there were no significant differences between the two groups in the severity of both BG-EPVS and WM-EPVS (*p*=0.038, see Table 1). Multivariate logistic regression was applied to determine risk factors for MCI patients, with vascular risk factors (history of hypertension, diabetes mellitus, hyperlipidemia, smoking and alcohol use), age, gender, the severity of WMHs, BG-EPVS and WM-EPVS. We found that severe WMHs were independent risk factors for MCI patients (*p*=0.013, 95%CI 0.23, 0.84, see Table 2). The result was consistent with the latest meta-analysis, which found WMHs have a medium-sized association with different cognitive functions in patients with MCI and a small, but statistically significant, association with cognition in Alzheimer's disease ([5](#_ENREF_5)). This may because white matter forms the dominant proportion of brain structure, which reflects the importance of connection and networks in neural structure and consequently brain function.


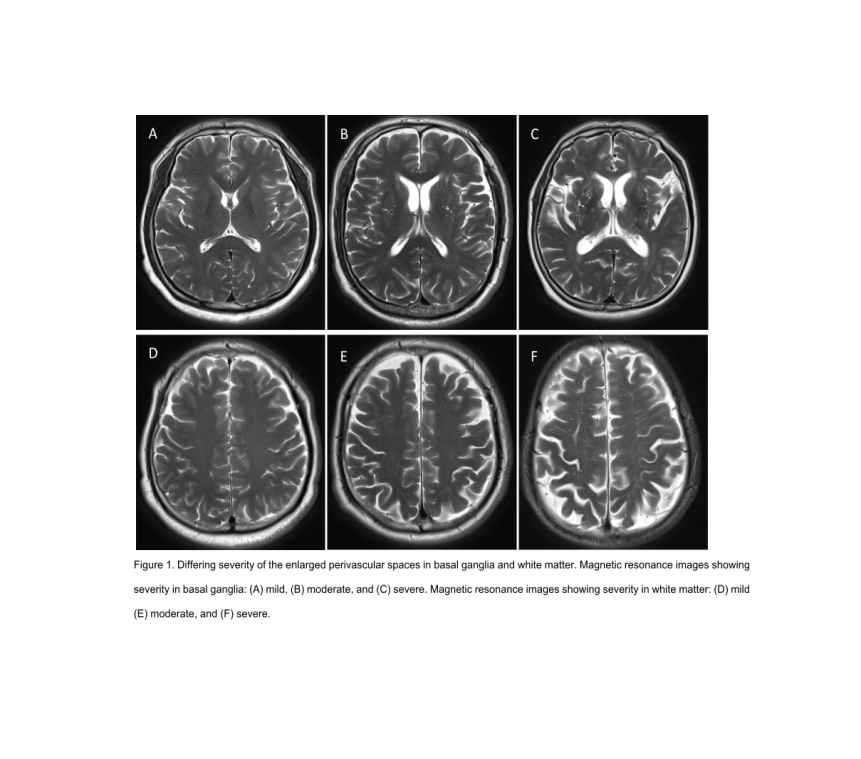

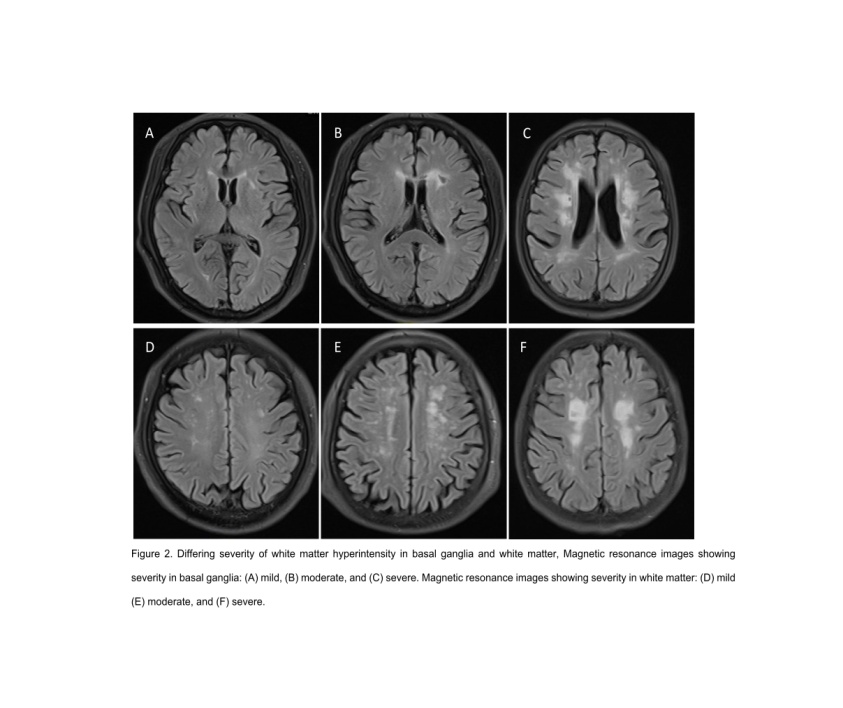


| Table 1 Comparisons of WMHs and EPVS between MCI and non-MCI patients. | | | | |
| --- | --- | --- | --- | --- |
|  | MCI group  N=48 | non-MCI group  N=34 | U/ x^2^ | *P* |
| Total-WMHs (Fazekas scale) |  |  | 6.552 | 0.038* |
| Mild (0–2) | 9(18.8%) | 13(38.2%) |  |  |
| Moderate (3–4) | 21(43.8%) | 16(47.1%) |  |  |
| Severe (5–6) | 18(37.5%) | 5(14.7) |  |  |
| BG-EPVS |  |  | 2.118 | 0.347 |
| Mild (0–1) | 23(47.9%) | 21(61.8%) |  |  |
| Moderate (2) | 12(25%) | 8(23.5%) |  |  |
| Severe (3–4) | 13(27.1%) | 5(14.7%) |  |  |
| WM-EPVS |  |  | 0.276 | 0.871 |
| Mild (0–1) | 38(79.2%) | 28(82.4%) |  |  |
| Moderate (2) | 4(8.3%) | 3(8.8%) |  |  |
| Severe (3–4) | 6(12.5) | 3(8.8%) |  |  |

WMHs= white matter hyperintensities, BG-EPVS=Basal ganglia enlarged perivascular space, WM-EPVS= white matter enlarged perivascular space, MCI= mild cognitive impairment. **P*<.05.

| Table 2 Logistic regression for relative factors associated with MCI patients | | | |
| --- | --- | --- | --- |
| Variables | OR | *p* value | 95% CI |
| WMHs (Severe） | 0.444 | 0.013 | (0.234-0.844) |

WMHs= white matter hyperintensities, OR=odds ratio.

**Reference**

1. Blair, G.W., Hernandez, M.V., Thrippleton, M.J., Doubal, F.N., and Wardlaw, J.M. Advanced Neuroimaging of Cerebral Small Vessel Disease. *Curr Treat Options Cardiovasc Med* (2017)19(7)**,** 56. doi: 10.1007/s11936-017-0555-1.

2. Doubal, F.N., MacLullich, A.M., Ferguson, K.J., Dennis, M.S., and Wardlaw, J.M. Enlarged perivascular spaces on MRI are a feature of cerebral small vessel disease. *Stroke* (2010)41(3)**,** 450-454. doi: 10.1161/STROKEAHA.109.564914.

3. Yang, S., Zhang, X., Yuan, J., Yin, J., and Hu, W. Serum Uric Acid is Independently Associated with Enlarged Perivascular Spaces. *Sci Rep* (2017)7(1)**,** 16435. doi: 10.1038/s41598-017-16715-5.

4. Zhang, X., Ding, L., Yang, L., Qin, W., Yuan, J., Li, S., et al. Brain Atrophy Correlates with Severe Enlarged Perivascular Spaces in Basal Ganglia among Lacunar Stroke Patients. *PLoS One* (2016)11(2)**,** e0149593. doi: 10.1371/journal.pone.0149593.

5. van den Berg, E., Geerlings, M.I., Biessels, G.J., Nederkoorn, P.J., and Kloppenborg, R.P. White Matter Hyperintensities and Cognition in Mild Cognitive Impairment and Alzheimer's Disease: A Domain-Specific Meta-Analysis. *J Alzheimers Dis* (2018)63(2)**,** 515-527. doi: 10.3233/JAD-170573.
